# Functions of J‐domain proteins in mitochondrial protein biogenesis

**DOI:** 10.1002/pro.70516

**Published:** 2026-03-02

**Authors:** Vitasta Tiku, Georg Bossenz, Janine Kirstein, Thomas Becker

**Affiliations:** ^1^ Institute of Biochemistry and Molecular Biology, University Hospital Bonn, University of Bonn Bonn Germany; ^2^ Leibniz‐Institute on Aging‐Fritz‐Lipmann‐Institute (FLI) Jena Germany; ^3^ Institute for Biochemistry and Biophysics, Friedrich‐Schiller‐Universität Jena Germany

**Keywords:** ER‐SURF, Hsp70, J‐domain protein, mitochondria, protein targeting, TOM complex

## Abstract

Mitochondrial biogenesis and functions depend on the import and assembly of more than 1000 proteins that are made as precursors on cytosolic ribosomes. The majority of these precursor proteins are transported from the ribosome to the translocase of the outer membrane (TOM complex), which constitutes the main entry site for mitochondrial precursors. The transient localization of mitochondrial precursor proteins in the cytosol represents a major burden for cellular proteostasis since these proteins can aggregate and accumulate in different cellular compartments, causing proteotoxic stress. Inside mitochondria, protein translocases sort the precursor proteins into the mitochondrial subcompartments—outer and inner membrane, the intermembrane space and matrix. The imported proteins have to be folded and efficiently assembled into functional protein complexes. Molecular chaperones such as Hsp70 monitor these processes to minimize proteotoxic stress. J‐domain proteins stimulate the ATPase activity of Hsp70 and recruit the chaperones to their clients in the biogenesis of mitochondrial proteins. They ensure protein targeting to mitochondria, drive protein import into mitochondria, as well as folding and assembly of mitochondrial proteins. Here, we summarize the emerging view of how J‐domain proteins guide mitochondrial precursor proteins from their synthesis in the cytosol until their folding into a mature protein and assembly into protein complexes in mitochondria.

## 1 INTRODUCTION

Mitochondria are double membrane‐bound cell organelles that emerged by an endosymbiotic event two billion years ago. A eukaryotic ancestor cell incorporated a prokaryotic cell, which is similar to nowadays alpha‐proteobacteria. Over the course of evolution most of the genetic information of the endosymbiont was either lost or transferred to the host genome. Only a small circular genome remains that encodes eight proteins in baker's yeast and 13 proteins in human mitochondria. Consequently, mitochondria import about 99% of their 1000 proteins in yeast and up to 1500 proteins in human cells (Bykov et al., 2020; Endo & Wiedemann, 2025; Morgenstern et al., 2017; Morgenstern et al., 2021; Pfanner et al., 2019). Protein import is essential to ensure that mitochondria carry out a magnitude of different biochemical functions like energy production, iron–sulfur cluster formation, lipid‐ and amino acid biosynthesis, metabolic steps in, for example, the urea cycle and cellular signaling in inflammation and apoptosis (Nunnari & Suomalainen, 2012; Spinelli & Haigis, 2018).

Most mitochondrial proteins are produced on cytosolic ribosomes and targeted to the mitochondrial surface. The consequence is that mitochondrial precursor proteins are transiently present outside mitochondria, which can be a major burden for the cell when protein import is impaired. Mitochondrial precursor proteins that fail to get imported into mitochondria can aggregate and/or mislocalize to different cellular compartments, causing proteotoxic stress in the cell (Krämer et al., 2023; Nowicka et al., 2021; Shakya et al., 2021; Wang & Chen, 2015; Wrobel et al., 2015). Molecular chaperones like Hsp70 and Hsp90 keep mitochondrial precursor proteins in an import‐competent state and guide them to the receptors of the translocase of the outer mitochondrial membrane (TOM complex) (Backes et al., 2021; Hoseini et al., 2016; Young et al., 2003). J‐domain proteins are co‐chaperones of Hsp70 proteins and stimulate their ATPase activity, facilitating their binding to client proteins. They play central functions in the Hsp70‐mediated targeting of precursor proteins to mitochondria (Becker et al., 2019; Bykov et al., 2020; Ruger‐Herreros et al., 2024).

The TOM complex forms the entry gate for most mitochondrial precursor proteins into mitochondria. Specific protein translocases mediate the further sorting of these proteins into the mitochondrial outer and inner membranes, the intermembrane space and matrix (Busch et al., 2023; Endo & Wiedemann, 2025; Kizmaz et al., 2024). The presequence translocase (TIM23 complex) transports proteins into or across the inner membrane. The precursor proteins contain an amino‐terminal presequence, which is cleaved off by the mitochondrial processing peptidase (MPP) upon import into mitochondria. Translocation into the matrix involves the mitochondrial Hsp70 (mtHsp70) that is also regulated by a J‐domain protein. The carrier translocase (TIM22 complex) inserts proteins with multiple transmembrane domains such as carrier proteins that lack a cleavable presequence. The mitochondrial intermembrane space import and assembly (MIA) pathway imports cysteine‐rich proteins into the intermembrane space. Finally, the sorting and assembly machinery (SAM) integrates and folds β‐barrel proteins into the outer membrane. These four import routes are present in both yeast and human mitochondria. In contrast, the insertion of α‐helical membrane proteins occurs via different proteins in yeast (MIM complex) and human (MTCH2) mitochondria (Doan et al., 2020; Guna et al., 2022; Papic et al., 2011).

J‐domain proteins are central players in mitochondrial protein biogenesis. They function both outside and inside mitochondria to control Hsp70 function and substrate specificity. Outside mitochondria, different J‐domain proteins guide different types of precursor proteins on their journey to mitochondria. Some of the J‐domain proteins display a broad substrate spectrum (generalists), while others recognize a few selected substrates (specialists). When protein import into mitochondria fails, J‐domain proteins are involved in the sequestration of non‐imported precursor proteins in protein deposits and in modulating cellular stress response pathways. Inside mitochondria, J‐domain proteins facilitate protein import via the TIM23 translocase, mediate protein folding and ensure efficient transfer of the imported protein into the assembly line to promote formation of protein complexes such as respiratory chain complexes. Here, we summarize the multitude of functions mediated by J‐domain proteins to reveal their central role for biogenesis and control of mitochondrial precursor proteins in baker's yeast *Saccharomyces cerevisiae* and mammals.

## 2 THE ARCHITECTURE AND FUNCTIONS OF CELLULAR J‐DOMAIN PROTEINS

**FIGURE 1:**
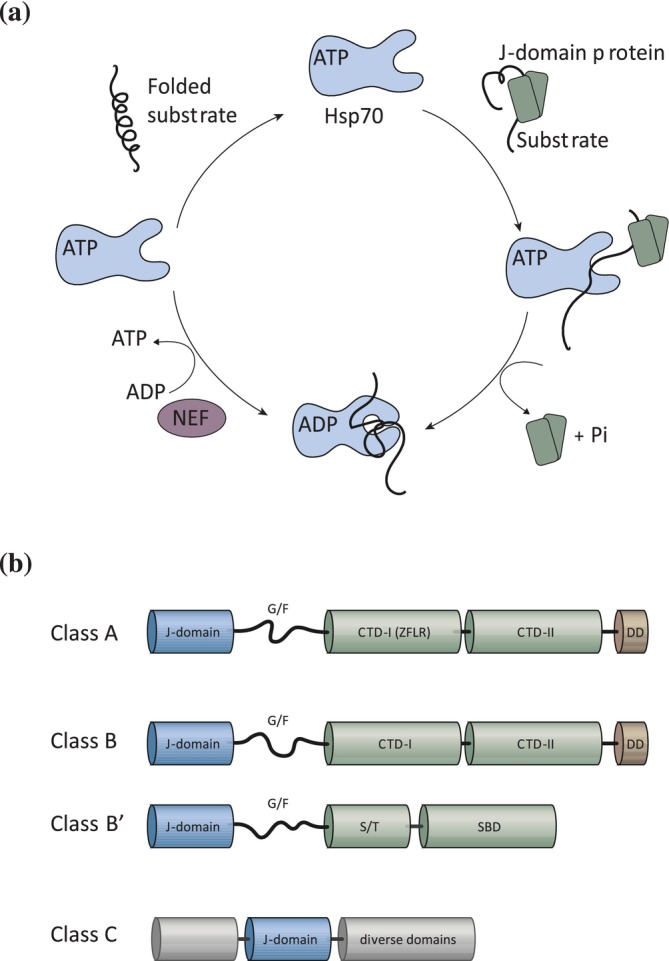
Functions and architecture of J‐domain proteins. (a) Role of J‐domain proteins in the Hsp70 reaction cycle of substrate binding and release. J‐domain proteins recruit Hsp70 chaperones to the client protein and subsequently stimulate the ATPase activity of the chaperone. Upon ATP‐hydrolysis, the Hsp70 undergoes a conformation change, leading to a closed state with a high affinity for substrates. The nucleotide exchange factor (NEF) stimulates the exchange of ADP with ATP of Hsp70, which results in its low affinity for substrates and their subsequent release. (b) Domain organization of J‐domain proteins of classes A, B, B′ and C. The J‐domain depicted in blue and located at the N‐terminus of class A and B J‐domain proteins. The J‐domain is followed by a flexible glycine/phenylalanine‐rich domain (G/F) that is depicted as a disordered region. The C‐terminal domains (CTD) are separated by a hinge region into CTD‐I and CTD‐II and are depicted in green. The dimerization domain (DD) present in class A and B J‐domain proteins is shown in brown. Class A J‐domain proteins contain a Zinc‐finger like region (ZFLR) in CTD‐I that is absent in class B J‐domain proteins. Non‐canonical class B members (class B′) have a more diverse structure including a serine/threonine rich region (S/T) followed by a C‐terminal substrate binding domain (SBD). The J‐domain of class C proteins can be located at different regions in the protein and can be flanked by diverse domains that are depicted in gray.

J‐domain proteins form the largest and most diverse class of molecular chaperones in eukaryotic cells (Brehme et al., 2014). J‐domain proteins are defined by the presence of a J‐domain that is approximately 70 aa residues long and includes the highly conserved HPD motif that is essential for Hsp70 interaction and stimulation of its ATPase activity (Mayer & Bukau, 2005). Hsp70 is an essential molecular chaperone that participates in numerous constitutive and stress‐responsive cellular pathways. Its functions include facilitating protein folding, preventing the accumulation of cytotoxic protein aggregates, supporting the import of polypeptides into subcellular compartments, promoting the assembly and disassembly of oligomeric protein complexes and directing irreversibly damaged proteins toward degradation pathways. Importantly, these diverse activities rely on J‐domain proteins which select the protein substrates and stimulate the Hsp70 ATPase that is needed for the nucleotide‐dependent conformational changes that govern substrate engagement of Hsp70. Cycling between ATP‐ and ADP‐bound states enables Hsp70 to alternately capture and release substrate proteins (Figure 1a) (Mayer & Bukau, 2005). J‐domain proteins bind a broad spectrum of unfolded, misfolded, or aggregated proteins and target them to their cognate Hsp70 chaperone (Kampinga et al., 2019; Kampinga & Craig, 2010). Nucleotide exchange factors restore the ground state of Hsp70 by promoting ADP dissociation that allows ATP rebinding and the release of the substrate protein (Bracher & Verghese, 2015).

Based on their domain composition and overall structural organization, J‐domain proteins can be grouped into three principal classes (Kampinga & Craig, 2010) (Figure 1b). Class A J‐domain proteins adopt the canonical architecture exemplified by DnaJ of *E. coli*. They contain an N‐terminal J‐domain, followed by a glycine‐ and phenylalanine‐rich (G/F) segment and two C‐terminal β‐sandwich domains and a zinc‐finger–like region (ZFLR) protrudes from the first C‐terminal domain (Cheetham & Caplan, 1998). Class B J‐domain proteins can be divided into two types, canonical and non‐canonical class B J‐domain proteins. Canonical class B J‐domain proteins share the C‐terminal substrate binding and dimerization domains with class A (Kirstein et al., 2025). The non‐canonical that are referred to as B′ J‐domain proteins do not possess these domains and have instead a serine/threonine rich domain followed by a substrate binding domain immediately after the G/F segment (Ayala Mariscal & Kirstein, 2021). All class B J‐domain proteins lack a ZFLR that is the defining feature of class A J‐domain proteins (Kirstein et al., 2025). The G/F region in both class A and class B J‐domain proteins is generally regarded as largely disordered (Hobbs et al., 2025). Many class A J‐domain proteins and a subset of class B J‐domain proteins form constitutive homodimers via their C‐terminal dimerization domains. It is not yet clear if the dimerization or potentially oligomerization that has been observed for the B′ J‐domain proteins is a prerequisite to bind their substrates or if these assemblies constitute a transient storage form (Kakkar et al., 2016; Scior et al., 2021). Class C are the largest sub‐family of the J‐domain proteins and comprise all J‐domain proteins that do not fit the criteria of classes A and B. They contain the name‐giving J‐domain that however can be located anywhere in the protein and otherwise show no resemblance to class A and B J‐domain proteins (Ayala Mariscal & Kirstein, 2021). Class C J‐domain proteins are very diverse in their domain architecture and contain, for example, ubiquitin‐binding domains, cysteine‐rich regions, GTP‐binding domains, tetratricopeptide repeats (TRPs) and clathrin‐binding domains to participate in very diverse biological processes (Kampinga et al., 2019; Kampinga & Craig, 2010). Interestingly, J‐domain proteins cannot only form homodimers, but also heterodimers of class A and B J‐domain proteins and thereby exert a synergistic effect on Hsp70 to potentiate the chaperone complex composed of Hsp70/Hsp110 and mixed class J‐domain proteins to disaggregate protein aggregates (Kirstein et al., 2017; Nillegoda et al., 2015).

**FIGURE 2:**
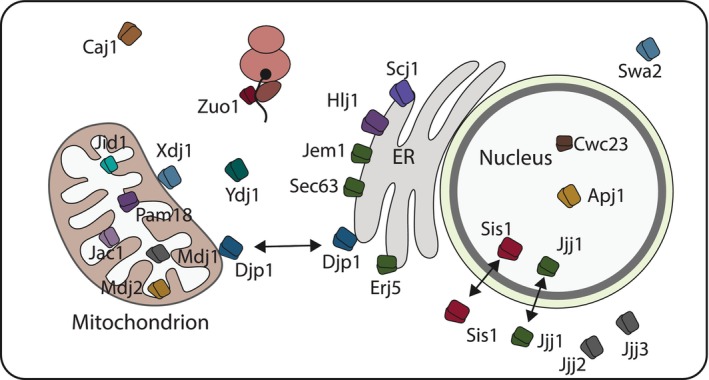
Localization of J‐domain proteins in the cell. Overview about the cellular localization of the 22 J‐domain proteins in yeast. Some J‐domain proteins (Sis1, Djp1, Jem1) localize to two different compartments as indicated.

In the course of evolution, the J‐domain protein family expanded both in number and structural diversity. In eukaryotic organisms, there is a more than two‐fold increase in the number of J‐domain proteins relative to their partner Hsp70 chaperones (Brehme et al., 2014). In yeast, 21 J‐domain proteins and 11 Hsp70 are present, whereas in human cells, 50 J‐domain proteins and 11 Hsp70 were identified (Malinverni et al., 2023; Ruger‐Herreros et al., 2024). J‐domain proteins have been found in the cytosol, ER, nucleus and mitochondria. Most of the J‐domain proteins localize to the cytosol/nucleus (34 in human and 14 in yeast), followed by those residing in the ER (9 in human and 3 in yeast) and those that are present within and associated with mitochondria (7 for both yeast and human) (Figure 2) (Malinverni et al., 2023). Additionally, several cytoplasmic J‐domain proteins function in the biogenesis of mitochondrial proteins, reflecting the central role of this protein family for mitochondrial function and biogenesis (Table 1).

**TABLE 1:** Listed are J‐domain proteins involved in mitochondrial protein biogenesis.

Cytosolic protein factor	Function	Known substrates	Localization	Organism	References
Ydj1 (yeast DnaJ)	Protein targeting	OM proteins (β‐barrel; Om14); IM proteins (cleavable precursors); IMS proteins; Matrix proteins	Cytosol	Yeast	(Caplan et al., 1992; Hoseini et al., 2016; Jores et al., 2018)
Apj1 (Anti‐Prion 1)	Protein aggregation and heat shock response	Precursors of mitoribosomal subunits and carrier proteins	Nucleus	Yeast	(Caplan et al., 1992; Hoseini et al., 2016; Jores et al., 2018)
Sis1 (Slt4 suppressor)	Protein targeting and protein sequestration	OM proteins (β‐barrel; Om14); IM proteins (cleavable precursors); IMS proteins; Matrix proteins	Cytosol	Yeast	(Hoseini et al., 2016; Jores et al., 2018)
Djp1 (DnaJ protein)	Protein targeting via ER‐SURF	OM proteins (β‐barrel; Om14; Mim1); IM proteins (cleavable precursors)	Cytosol	Yeast	(Hansen et al., 2018; Opaliński et al., 2018)
Xdj1 (DnaJ protein)	Protein transport at the TOM complex	OM proteins (Tom22); IM proteins (cleavable precursors)	Cytosol and mitochondria	Yeast	(Opaliński et al., 2018)
Pam18 (presequence translocase‐associated motor)	Protein import and assembly	Presequence‐containing proteins	Mitochondrial inner membrane	Yeast	(D'Silva et al., 2003; Mokranjac et al., 2003; Truscott et al., 2003)
Mdj1 (Mitochondrial DnaJ 1)	Protein folding	Matrix proteins	Mitochondrial matrix	Yeast	(Horst et al., 1997; Rowley et al., 1994; Westermann et al., 1996)
Mdj2 (Mitochondrial DnaJ 2)	Protein import	Presequence‐containing proteins	Mitochondrial inner membrane	Yeast	(Mokranjac et al., 2005)
Jac1 (J‐type accessory chaperone)	Iron–sulfur protein biogenesis	Iron–sulfur cluster proteins	Matrix	Yeast	(Lill & Freibert, 2020)
DNAJA1 (DnaJ homolog subfamily A member 3)	Protein targeting; stress response	IM proteins (carrier)	Cytosol	Mammals	(Bhangoo et al., 2007)
DNAJA2	Protein targeting	IM proteins (carrier)	Cytosol	Mammals	(Bhangoo et al., 2007)
DNAJA3	Protein folding	Matrix proteins	Matrix	Mammals	(Shin et al., 2021)
DNAJA4	Protein targeting	IM proteins (carrier)	Cytosol	Mammals	(Bhangoo et al., 2007)
DNAJC11	Associates with SAM complex	Unknown	Mitochondrial outer membrane	Mammals	(Xie et al., 2007)
DNAJC15	Protein import	Presequence‐containing proteins	Mitochondrial inner membrane	Mammals	(Kroczek et al., 2026; Richter‐Dennerlein et al., 2014)
DNAJC19	Protein import	Presequence‐containing proteins	Mitochondrial inner membrane	Mammals	(Kroczek et al., 2026; Richter‐Dennerlein et al., 2014)
DNAJC24	Protein targeting	Outer membrane proteins	Cytosol	Mammals	(Muthukumar et al., 2024)
DNAJC30	Turnover of the N‐module of complex I	F1FO‐ATP synthase and complex I	Inner membrane	Mammals	(Stenton et al., 2021; Tebbenkamp et al., 2018)
HSC20	Iron–sulfur protein biogenesis	Iron–sulfur cluster proteins	Matrix	Yeast	(Lill & Freibert, 2020)

*Note*: Depicted are the localizations and functions of the J‐domain proteins.

Abbreviations: IM, inner membrane; IMS, intermembrane space; OM, outer membrane.

## 3 J‐DOMAIN PROTEINS FUNCTIONS IN PROTEIN TARGETING TO MITOCHONDRIA

### 3.1 Pathways of protein targeting and import into mitochondria

**FIGURE 3:**
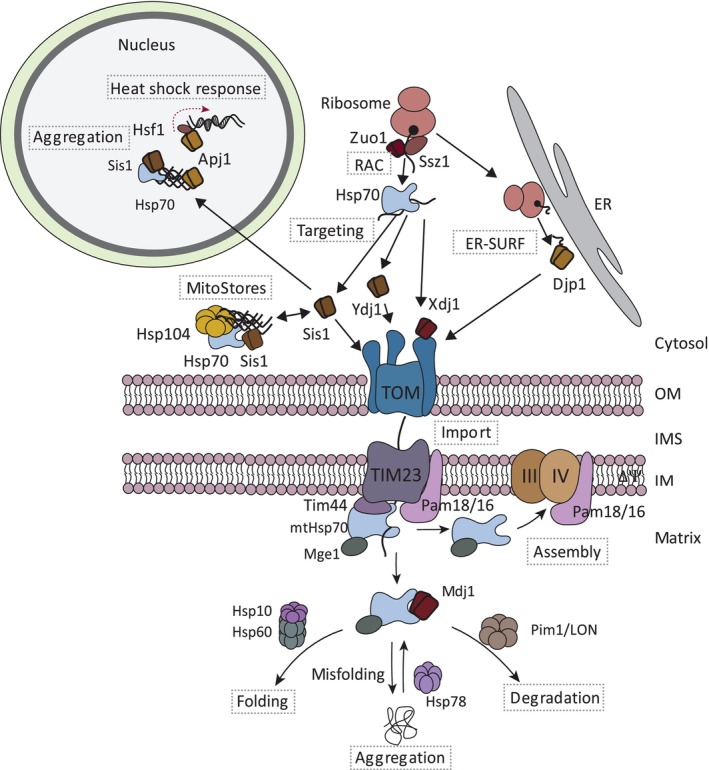
Overview about the functions of J‐domain proteins in the biogenesis and quality control of mitochondrial protein targeting. Cytosolic ribosomes produce the bulk of mitochondrial precursor proteins. The ribosome‐associated complex (RAC) consists of the J‐domain protein Zuo1 and its partner protein Ssz1 and cooperates with cytosolic Hsp70 proteins during early folding steps at the ribosome. Hsp70 proteins target different types of mitochondrial precursor proteins to the receptors of the translocase of the outer membrane (TOM complex). The targeting is assisted by cytosolic J‐domain proteins (Ydj1, Sis1, and Xdj1). Xdj1 binds directly to the TOM complex and promotes import into mitochondria. Some mitochondrial precursor proteins are first produced on the surface of the endoplasmic reticulum and are subsequently guided by Djp1 to mitochondria (ER‐SURF pathway). Sis1 and Apj1 are involved in sequestration of non‐imported mitochondrial precursor proteins into protein deposits in the cytosol (MitoStores) or the nucleus, respectively. Apj1 associates with the heat shock factor 1 (Hsf1) to modulate the heat shock response in response to accumulation of precursor proteins. Inside mitochondria, the presequence translocase (TIM23 complex) mediates the membrane potential (∆Ψ)‐dependent transport of presequence‐containing proteins across or into the inner membrane. The mitochondrial processing peptidase removes the presequence to allow subsequent folding steps (not shown). The TIM23 complex cooperates with the PAM module to transport precursor proteins into the mitochondrial matrix. The core subunit of the PAM module is the mitochondrial Hsp70 (mtHsp70) that docks to the TIM23 complex via Tim44. The J‐domain protein Pam18, together with its partner protein Pam16 and the nucleotide exchange factor Mge1, associates with the TIM23 complex to control the mtHsp70 activity during protein import. The Pam18/16 module also associates with the respiratory chain complexes to mediate their assembly. The mtHsp70 exists a second major pool together with the J‐domain proteins Mdj1 and Mge1. This pool mediates the folding of precursor proteins with or without the chaperonin Hsp60. mtHsp70 cooperates with Hsp78 in unfolding of aggregates proteins and with the protease Pim1/LON in protein degradation.

Protein transport of mitochondrial precursor proteins toward the receptors of the TOM complex can occur via different mechanisms. Co‐ and post‐translational protein import as well as transport via the surface of the endoplasmic reticulum (ER‐SURF) to mitochondria have been reported (Figure 3). The co‐translational protein import involves coupled protein biosynthesis and import and was reported for various mitochondrial precursor proteins (Becker et al., 2019; Bykov et al., 2020). Mitochondria‐directed ribosome profiling suggest that particularly hydrophobic inner membrane proteins could follow this pathway in yeast (Williams et al., 2014). Recent screening approaches based on mitochondria‐specific ribosome profiling in mammalian cells suggest that about 20% of mitochondrial proteins are imported in a co‐translational manner (Lapointe et al., 2018; Luo et al., 2025; Zhu et al., 2025). Similarly, it has been estimated that up to one third of the mitochondrial proteins follow a co‐translational import pathway in yeast (Lapointe et al., 2018; Williams et al., 2014). Analysis of the identified substrates indicated that particularly multi‐domain proteins are imported co‐translationally (Zhu et al., 2025). Supporting this view, it was reported that the amino‐terminal presequence binds to the Tom20 receptor once it emerges from the ribosome (Eliyahu et al., 2012). In this model, it is critical that the translating ribosomes are in close proximity to the TOM complex (Gold et al., 2017). In yeast, precursors of some hydrophobic inner membrane proteins are first targeted to the surface of the ER, which is termed the ER‐SURF pathway (Hansen et al., 2018; Koch et al., 2024). These proteins are synthesized at the ER and subsequently transported to mitochondria. The ER appears to constitute a scaffold to keep these hydrophobic proteins in solution (Hansen et al., 2018). According to current views, the vast majority of mitochondrial precursor proteins are imported in a post‐translational manner. Here, the precursor proteins are first synthesized by cytosolic ribosomes and subsequently guided by molecular chaperones to TOM receptors (Hansen et al., 2018). J‐domain proteins play a central role in post‐translational protein import and the ER‐SURF pathway as outlined below.

### 3.2 Functions of J‐domain proteins in the targeting of precursor proteins to the mitochondrial surface

The transport of precursor proteins to the mitochondrial surface is facing several challenges. First, the precursors have to be kept in an unfolded state and their premature folding or misfolding must be prevented. The TOM pore formed by the β‐barrel of Tom40 can only accommodate partially folded proteins like α‐helical hairpin structures but not transport fully folded proteins (Wiedemann et al., 2001). Second, many precursor proteins can form aggregates or mislocalize to different cellular compartments when they are not efficiently imported (Backes et al., 2021; Krämer et al., 2023; Shakya et al., 2021). Third, mitochondrial precursor proteins display different biochemical and biophysical properties like proteins with transmembrane domains, cysteine‐rich domains, β‐barrel proteins, soluble proteins or proteins that are part of multi‐subunit protein complexes. All these proteins are transiently present in the cytosol, which represents a challenge for the cellular proteostasis network. How does the cell cope with these different precursor proteins? Molecular chaperones like Hsp70 and Hsp90 bind to a large variety of mitochondrial precursor proteins to maintain their import‐competent state (Becker et al., 2019; Bykov et al., 2020). They guide precursor proteins from the ribosome to the receptors of the TOM complex on mitochondria. The TOM complex contains two major receptor proteins, Tom20 and Tom70. While Tom20 is the major receptor for proteins with a cleavable presequence, Tom70 binds predominantly to hydrophobic and aggregation‐prone precursor proteins (Backes et al., 2021). However, both receptor proteins display overlapping substrate specificity. The Tom70 receptor forms a clamp‐type tetratricopeptide (TPR) domain that binds to the carboxy‐terminus of Hsp70 and Hsp90 (Young et al., 2003). Tom70 is important for the import of aggregation‐prone proteins such as carrier proteins. An outer membrane‐anchored TPR‐domain can partially compensate for the loss of Tom70, reflecting its central role for Tom70 function (Backes et al., 2021).

J‐domain proteins play a critical role in all steps of targeting of precursor proteins to the mitochondrial surface (Figure 3). They function as co‐chaperones of Hsp70 via recruiting the chaperone to the substrates and stimulating the ATPase activity to facilitate binding of Hsp70 to the client proteins (Hipp et al., 2019; Kampinga & Craig, 2010; Ruger‐Herreros et al., 2024). Thereby, J‐domain proteins function in all steps of mitochondrial protein import, including ribosome‐associated protein folding, transport through the cytosol and docking onto TOM receptors. In yeast, the cooperation of the ribosome‐associated complex (RAC) with two closely related Hsp70 proteins (Ssb1/Ssb2) plays an important role in the folding of nascent polypeptides emerging at the ribosomal exit tunnel. The RAC complex is conserved from mammals to yeast and consists of the J‐domain protein Zuo1 (also termed Zuotin) and its associated partner Ssz1 (Kaschner et al., 2015; Lee et al., 2021). Ssz1 is an Hsp70‐like protein but it neither hydrolyzes ATP nor binds substrate proteins. The Ssz1‐Zuo1 complex binds to translating ribosomes and stimulates the ATPase activity of a second Hsp70 chaperone, Ssb1/Ssb2, which delays folding of the nascent polypeptide until the complete protein domain is produced at the ribosomes (Huang et al., 2005). Ssb1/Ssb2 binds to a large set of substrates including several mitochondrial proteins, reflecting its general function in early steps of protein folding (Döring et al., 2017; Stein et al., 2019). In the absence of either Ssb1/Ssb2 or RAC, newly synthesized proteins including mitochondrial proteins form aggregates (Willmund et al., 2013). Interestingly, Zuo1 has been recently linked to mitochondria‐induced stress. The double deletion of Tom70 and its paralog Tom71 leads to slow growth and defects in mitochondrial biogenesis. Parallel loss of Zuo1 partially restores slow growth and translational rates upon heat shock (Qian et al., 2025). The underlying molecular mechanisms remain unknown.

Cytosolic J‐domain proteins have been implicated in the transport of mitochondrial proteins toward the mitochondrial surface (Becker et al., 2019; Ruger‐Herreros et al., 2024). Ydj1 and Sis1 with about 40.000 and 28.000 copies per cell, respectively, are the most abundant J‐domain proteins in yeast (Ruger‐Herreros et al., 2024). Experimental evidence reveals an involvement of these J‐domain proteins in the targeting of proteins to mitochondria. First, binding studies with yeast translational extract demonstrated that the two highly abundant J‐domain proteins, Ydj1 and Sis1, bind to various mitochondrial precursor proteins, including presequence‐containing precursor proteins, inner membrane and intermembrane space proteins, β‐barrel and α‐helical anchored outer membrane proteins (Coyne et al., 2023; Drwesh et al., 2022; Hoseini et al., 2016; Jores et al., 2018). Second, Ydj1 and Sis1 could be cross‐linked to β‐barrel proteins, demonstrating that they directly interact with their substrates. The parallel knock down of both Ydj1 and Sis1 results in impaired import of β‐barrel proteins into mitochondria (Jores et al., 2018), pointing to a central and partly redundant role of these J‐domain proteins in targeting of β‐barrel proteins to mitochondria. Third, the biogenesis of presequence‐containing precursor proteins is impaired in *ydj1* mutant cells (Atencio & Yaffe, 1992; Caplan et al., 1992). Finally, Ydj1 and Sis1 from yeast cytosolic extracts can bind to the cytosolic domain of Tom20 and to a lesser extent to the cytosolic domain of Tom70 (Hoseini et al., 2016). Altogether, these observations indicate that Ydj1 and Sis1 are involved in the targeting of a variety of different mitochondrial precursor proteins to TOM receptors.

Two further cytosolic J‐domain proteins, Xdj1 and Djp1, are involved in mitochondrial protein biogenesis. The copy numbers of Djp1 (about 5600) and Xdj1 (about 1800) are lower compared to those of Ydj1 and Sis1 (Ruger‐Herreros et al., 2024) and both J‐domain proteins exhibit more specialized functions. Using genetic screens and import assays in semi‐intact cells, Djp1 was identified as a factor of the ER‐SURF pathway. Here, it transfers hydrophobic precursor proteins like Oxa1 to mitochondria (Hansen et al., 2018). Microscopic analysis revealed that GFP‐fused Djp1 mainly localized to the ER. In the absence of Djp1, GFP‐fused Oxa1 accumulated at the ER, pointing to a crucial function of Djp1 in the transfer of hydrophobic precursor proteins to mitochondria in the ER‐SURF pathway (Hansen et al., 2018). Proteins involved in contact sites between mitochondria and the ER such as the ER mitochondria encounter structure (ERMES) and Tom70 facilitate the transfer of precursor proteins toward mitochondria (Koch et al., 2024). Proteomic analysis revealed that the ER‐SURF substrates are mainly hydrophobic inner membrane proteins that accumulate at the ER when the contact sites are disrupted (Koch et al., 2024). Remarkably, recombinantly expressed Djp1 binds to the cytosolic domain of Tom70 (Opaliński et al., 2018), indicating that the interplay between both proteins could facilitate the transfer of precursor proteins to the TOM complex. Similarly, deletion of Djp1 leads to accumulation of the outer membrane protein Mim1 at the ER, indicating that Mim1 could be transported via the ER‐SURF pathway (Papic et al., 2011). Another J‐domain protein, Xdj1, plays an important role for the biogenesis of Tom22, but not for Mim1 (Opaliński et al., 2018). Xdj1 is also involved in the transfer of precursor proteins to the TOM complex. It binds to hydrophobic precursor proteins as well as to the Tom22 receptor via its substrate binding domain, suggesting that the receptor domain of Tom22 replaces the precursor protein from the substrate binding domain of Xdj1 to allow the import of the precursor protein (Opaliński et al., 2018). While these interactions occur independently of Hsp70, the import of both Tom22 and mitochondrial precursor proteins depend on a functional J‐domain of Xdj1 and therefore on the interplay of Xdj1 with Hsp70 (Opaliński et al., 2018).

### 3.3 Functions of J‐domain proteins in protein targeting to mammalian mitochondria

In mammalian cells, the role of J‐domain proteins in the transport of precursor proteins to mitochondria is less defined. The cytosolic J‐domain proteins DNAJA1, DNAJB1 and DNAJB2 of the rabbit reticulocyte lysate stimulate the import of the ornithine transcarbamylase into isolated mitochondria (Terada et al., 1997) and an antibody against DNAJ1 inhibits its import (Kanazawa et al., 1997). Affinity purification of carrier proteins of reticulocyte lysate reveal that several chaperones bind to these hydrophobic proteins including Hsp70, Hsp90 and their co‐chaperones HOP, HIP and three J‐domain proteins DNAJA1, DNAJA2 and DNAJA4 (Bhangoo et al., 2007). A function of Hsp70 and Hsp90 in the import of carrier proteins into mammalian mitochondria has been described (Young et al., 2003). The J‐domain proteins bind to the carrier proteins, independently of their J‐domain. Remarkably, binding of J‐domain proteins lacking the J‐domain decreases the association of Hsp70 to precursors of carrier proteins and block their subsequent import into mitochondria, pointing to the central role of J‐domain proteins for the import of carrier proteins (Bhangoo et al., 2007). Finally, a split‐GFP reporter‐based screen revealed that in the absence of the cytosolic DNAJC24 the mitochondrial localization of model signal‐anchored and a multi‐spanning outer membrane protein is reduced (Muthukumar et al., 2024). Altogether, six cytosolic J‐domain proteins have been implicated to promote targeting of mitochondrial precursor proteins, however, molecular mechanisms such as interaction with TOM receptors and substrate specificity remain to be defined.

## 4 FUNCTION OF J‐DOMAIN PROTEINS IN MITOCHONDRIAL IMPORT STRESS

### 4.1 Role of J‐domain proteins in sequestration of precursor proteins in deposits

The import of mitochondrial precursor proteins can fail when precursor proteins prematurely fold or misfold in the cytosol or upon defects of the protein import apparatus. The depletion of the membrane potential that is generated by the activity of the respiratory chain can also affect protein transport into or across the inner membrane (Boos et al., 2019; Mårtensson et al., 2019; Pfanner et al., 2025; Wang & Chen, 2015; Wrobel et al., 2015). All these scenarios can cause clogging of the TOM complex by mitochondrial precursor proteins and in turn lead to their accumulation in the cytosol, which is eventually lethal for the cell (Wang & Chen, 2015; Wrobel et al., 2015). Non‐imported precursor proteins mislocalize to different compartments like the cytosol, nucleus, ER, or can be sorted to protein deposits such as MitoStores (den Brave et al., 2020; Krämer et al., 2023; Nowicka et al., 2021; Shakya et al., 2021). MitoStores are temporary stores of particularly presequence‐containing proteins. When the protein import stress ends, proteins can be extracted from the MitoStores by Hsp104 and delivered for protein import (Krämer et al., 2023). Hsp104 is a hexameric chaperone that resolves aggregated or misfolded proteins (Hipp et al., 2019). Molecular mechanisms remove precursors that are stalled at the TOM complex during import to deliver them for proteasomal degradation and thereby minimize the stress situations (Mårtensson et al., 2019; Weidberg & Amon, 2018). Whether or not J‐domain proteins are involved in these quality control pathways remains largely unknown. However, the yeast J‐domain protein Sis1 and the Hsp70, Ssa1, have been implicated in the degradation of unstable outer membrane proteins (Metzger et al., 2020). Furthermore, Sis1 has been found as a component of MitoStores (Krämer et al., 2023). Since it enables disaggregation of protein aggregates by acting as a co‐chaperone of the cytosolic Hsp70, Ssa1, and Hsp104 (Hipp et al., 2019; Kampinga & Craig, 2010; Ruger‐Herreros et al., 2024), Sis1 could also control aggregation/disaggregation of mitochondrial precursor proteins. Several non‐imported mitochondrial precursor proteins mislocalize to the nucleus (Shakya et al., 2021). In the nucleus, the J‐domain protein Apj1 binds to protein aggregates and together with Ssa1 mediates protein disaggregation, delivering them for proteasomal degradation. Remarkably, Hsp70‐Apj1 disaggregate proteins independently of Hsp104 (den Brave et al., 2020). Analysis of the binding partners of Apj1 revealed that mitochondrial precursor proteins like mitoribosomal subunits are present in these aggregates (den Brave et al., 2020; Flohr et al., 2025). Mitochondrial proteins are enriched at Apj1 upon blocking protein import or inhibition of the proteasome (den Brave et al., 2020; Flohr et al., 2025), indicating an important function of Apj1 in quality control of mitochondrial proteins. Interestingly, Apj1 binds to the heat shock transcription factor Hsf1. Hsf1 is released from Hsp70 upon heat shock to induce the expression of genes involved in the heat shock response (den Brave et al., 2020; Flohr et al., 2025). The heat shock response is prolonged in the absence of Apj1, revealing that Apj1 attenuates the heat shock response by binding to Hsf1 (Ruger‐Herreros et al., 2024). Thus, Apj1 links protein quality control to the regulation of the heat shock response. Clogging of the TOM complex with a precursor protein leads to the induction of the heat shock response and the down regulation of OXPHOS subunits (Boos et al., 2019). Whether the transcriptional response upon clogging of the TOM complex is linked to Apj1 remains to be demonstrated.

In mammalian cells, misfolding of mitochondrial proteins can induce the mitochondrial unfolded stress response (mitoUPR) (Horibe & Hoogenraad, 2007; Quirós et al., 2017; Shpilka & Haynes, 2018). In the mitoUPR, the expression of mitochondrial chaperones and proteases are induced to counteract the accumulation of misfolded proteins. A recent study uncovered that the cytosolic J‐domain protein DNAJA1 plays a central role in the stress response to mitochondrial protein misfolding (Sutandy et al., 2023). Here, the mitochondrial Hsp90 was inhibited to induce protein misfolding inside mitochondria, which in turn led to the production of reactive oxygen species (ROS) and the accumulation of mitochondrial precursor proteins in the cytosol. The cytosolic DNAJA1 integrates both stress signals to induce a transcriptional reprogramming leading to enhanced expression of genes encoding chaperones and proteasomal subunits (Sutandy et al., 2023). Defective mitochondria can release ROS into the cytosol that can oxidize two cysteine residues in its Zn‐finger‐like domain of DNAJA1 (Sutandy et al., 2023). Oxidized DNAJA1 has a higher affinity to Hsp70 and thereby promotes the recruitment of Hsp70 to accumulating mitochondrial precursor proteins that fail to get imported when mitochondria encounter stress (Sutandy et al., 2023). The redirection of Hsp70 to protein substrates releases HSF1 from the inhibitory complex with Hsp70. The released HSF1 can translocate to the nucleus to induce the expression of mitochondrial chaperones and proteases of the mitoUPR (Sutandy et al., 2023).

## 5 J‐DOMAIN PROTEINS PROMOTE IMPORT AND ASSEMBLY OF MITOCHONDRIAL PROTEINS

The TIM23 complex cooperates with the PAM module in protein translocation across the inner membrane into the mitochondrial matrix (Figure 3). The central subunit of the PAM module is the mitochondrial Hsp70 (mtHsp70) that drives by a cycle of binding and releasing of precursor proteins their transport into the matrix. ATP loading and hydrolysis also controls the binding of mtHsp70 to Tim44, which forms the docking site for mtHsp70 at the presequence translocase (Liu et al., 2003; Rassow et al., 1994; Schneider et al., 1994). The activity of mtHsp70 is regulated by co‐chaperones. The soluble Mge1 mediates the exchange of ADP to ATP and thereby substrate release. The J‐domain protein Pam18 (also termed Tim14) associates with the TIM23 complex and stimulates the ATP‐hydrolysis of mtHsp70 and thereby accelerates the chaperone‐mediated import of precursor proteins (D'Silva et al., 2003; Mokranjac et al., 2003; Truscott et al., 2003). Pam18 is anchored into the inner membrane and exposes a J‐domain into the matrix. The amino‐terminal intermembrane space domain interacts with the carboxy‐terminal domain of Tim17 of the TIM23 complex (Chacinska et al., 2005). Recent biochemical and structural analysis revealed that Tim17 forms a cavity to allow transport of precursor protein across and into the inner membrane (Fielden et al., 2023; Sim et al., 2023). When Pam18 is carboxy‐terminally fused to Tim17, it blocks lateral release of precursor proteins, indicating that the transmembrane domain of Pam18 could modulate protein transport into the inner membrane (Schendzielorz et al., 2018). Pam18 forms a stable heterodimer with Pam16 (also termed Tim16) (Mokranjac et al., 2006). Pam16 contains a J‐like domain, but is not capable to stimulate the ATPase activity of mtHsp70 because it lacks a HPD motif (Frazier et al., 2004). Instead, addition of Pam16 blocks the stimulatory effect of Pam18 on the ATPase activity of mtHsp70 in vitro (Li et al., 2004). This observation indicates that Pam16 has a regulatory function on the Pam18‐stimulated Hsp70 reaction cycle. Pam16 is further critical for stable binding of Pam18 to the presequence translocase (Frazier et al., 2004; Kozany et al., 2004). Pam16 and Pam18 are dynamically recruited to the translocating TIM23 complex to drive efficient protein import (Schulz et al., 2015). In yeast, a second membrane‐bound J‐domain protein, Mdj2, exists in the mitochondrial inner membrane, which also binds to Pam16. Overexpression of Mdj2 can partially compensate for the loss of Pam18 (Mokranjac et al., 2005). However, deletion of Mdj2 does not impair growth of the cell, indicating that Pam18 is the crucial J‐domain protein at the TIM23 complex. Similarly, two J‐domain proteins, DNAJC15 and DNAJC19, associate with the TIM23 translocase in human mitochondria (Kroczek et al., 2026; Richter‐Dennerlein et al., 2014). Depletion of DNAJC15, and to a lesser extent DNAJC19, impairs import of mitochondrial proteins including OXPHOS subunits leading to their accumulation in other cellular components and proteotoxic stress (Kroczek et al., 2026). Both J‐domain proteins bind MAGMAS, which is the functional homolog of Pam16 (Sinha et al., 2016). Like its yeast counterpart, MAGMAS controls the stimulating activity of both J‐domain proteins on mtHsp70 (Sinha et al., 2016). Interestingly, the mammalian J‐domain protein, DNAJC11, localizes to the outer mitochondrial membrane, where it associates with the SAM complex and components of the mitochondrial inner membrane and cristae organizing system (MICOS) (Xie et al., 2007). Supporting a functional role of the interaction of DNAJC11 with MICOS, a splice variant of DNAJC11 displayed altered cristae formation (Ioakeimidis et al., 2014). Interestingly, the Armadillo repeat containing protein 1 (ARMC1) associates with this protein complex to modulate mitochondrial distribution in the cell (Wagner et al., 2019). DNAJC11 mediates the release of AMRC1 from the mitochondrial outer membrane (McKenna et al., 2025). Whether or not DNAJC11 functions in protein import via the SAM complex remains to be studied.

### 5.1 Role of the J‐domain proteins in the formation of mitochondrial protein complexes

The J‐domain protein Pam18 appears to modulate the assembly of respiratory chain complexes. Pam16 and Pam18 also bind to respiratory chain supercomplexes consisting of complexes III and IV (Priesnitz et al., 2022; Wiedemann et al., 2007). Pam16 controls the segregation of Pam18 between the TIM23 and respiratory chain complexes (Figure 3). In a temperature sensitive mutant of Pam16 that is impaired in binding to Pam18, Pam18 is redistributed to the respiratory chain supercomplexes where it forms a homodimer (Priesnitz et al., 2022). Under these conditions, the assembly of complex IV subunits like Cox4 is increased (Priesnitz et al., 2022). Cox4 is essential for the formation of mature complex IV (Coyne et al., 2007; Frazier et al., 2004). Non‐assembled Cox4 binds to mtHsp70 (Figure 3), which could represent an important resource for Cox4 to allow rapid formation of complex IV (Böttinger et al., 2013). The mtHsp70‐bound pool of Cox4 is reduced and its assembly increased when Pam18 accumulates at the respiratory chain, pointing to a crucial role of the interplay of Pam18 and mtHsp70 in the assembly of complex IV (Priesnitz et al., 2022). Similarly, mammalian DNAJC15 interacts with assembly factors and subunits of the respiratory chain (Kroczek et al., 2026). Furthermore, upon depletion of DNAJC19 or DNAJC15, the cristae architecture and respiratory chain activity is modulated (Hatle et al., 2013; Janz et al., 2024). However, the molecular function of DNAJC15 in the formation of respiratory chain components remains to be investigated. The inner membrane DNAJC30 binds to the F_1_F_O_‐ATP synthase and may function to modulate the turnover of subunits of the N‐module of respiratory chain complex I (Stenton et al., 2021; Tebbenkamp et al., 2018). The molecular mechanism of DNAJC30 in this process remains to be determined. Finally, the human DNAJC19 binds to prohibitins in the inner membrane (Richter‐Dennerlein et al., 2014). Prohibitins form large ring‐like structures that constitute a molecular scaffold. The molecular interaction of DNAJC19 to prohibitins appears to be important for lipid remodeling (Richter‐Dennerlein et al., 2014). Altogether, the J‐domain proteins associate with additional protein machineries, indicating a functional versatility of Pam18 and its homologs in the organization of the inner mitochondrial membrane in mammalian cells.

## 6 THE MATRIX LOCALIZED J‐DOMAIN PROTEINS EXHIBIT MULTIPLE FUNCTIONS

The mtHsp70 associates with the TIM23 complex and forms a second population in the matrix of yeast mitochondria to fold imported proteins (Horst et al., 1997) (Figure 3). Both pools are characterized by their respective J‐domain protein. While mtHsp70 is controlled by Pam18 during protein import, the J‐domain protein Mdj1 stimulates the ATPase activity of Hsp70 during protein folding in the matrix (Horst et al., 1997; Rowley et al., 1994). Mdj1 does not function in protein import but stimulates protein folding of imported proteins inside mitochondria and promotes the removal of misfolded proteins in the matrix (Rowley et al., 1994; Wagner et al., 1994). The mammalian homolog DNAJA3 (Tid1) exists in two splice forms that constitute a heterodimer (Lu et al., 2006). DNAJA3 has been implicated in protein folding and removal of misfolded proteins in the mitochondrial matrix (Shin et al., 2021). Mdj1 is a versatile protein with different functions. It prevents aggregation of firefly luciferase that has been imported into mitochondria (Prip‐Buus et al., 1996). Overexpression of Mdj1 was reported to modulate early assembly steps of respiratory chain complexes (Pierrel et al., 2007). The J‐domain protein was found in complex with mitoribosome‐bound nascent polypeptides to stimulate their folding (Westermann et al., 1996). Considering that mtHsp70 binds to unassembled subunits of the cytochrome *c* oxidase and the ATP synthase (Böttinger et al., 2013; Song et al., 2023), one could speculate that a cooperation of Mdj1 and mtHsp70 could also control formation of protein assemblies. However, further studies are required to define the molecular mechanisms. Thus, Mdj1 promotes broad activity of mtHsp70 in mitochondrial proteostasis. Mdj1 also appears to fulfill specific functions. The interaction of Mdj1 with mitochondrial nucleoids (Ciesielski et al., 2013) was confirmed in Mdj1 temperature sensitive mutants where mitochondrial DNA polymerase activity was reduced and the mitochondrial inheritance was affected (Duchniewicz et al., 1999). Similarly, the mammalian homolog DNAJA3 binds to nucleoids as well and promotes integrity of mitochondrial DNA (Lu et al., 2006; Ng et al., 2014). These studies indicate that Mdj1 promotes maintenance of mitochondrial DNA. In yeast, two additional J‐domain proteins are present in the matrix and inner membrane. Jac1 (HSC20 in mammals) controls the function of the Hsp70, Ssq1, in iron–sulfur cluster protein biogenesis (Lill & Freibert, 2020). Jid1 was reported to localize to the inner membrane with a similar topology like Pam18 and Mdj2 (Bursać & Lithgow, 2009). However, a link to protein import was not found and the function of Jid1 remains elusive (Bursać & Lithgow, 2009). Altogether, mitochondrial J‐domain proteins exhibit a plethora of different functions for the biogenesis of mitochondrial proteins in the matrix and inner membrane.

## 7 J‐DOMAIN PROTEINS AND THEIR ROLE IN MITOCHONDRIA‐ASSOCIATED PATHOLOGIES

Over the past decade, mutations in nuclear‐encoded mitochondrial J‐domain proteins have emerged as causes of human mitochondrial diseases. Two J‐domain proteins, *DNAJC19* and *DNAJC30*, are now recognized as bona fide monogenic mitochondrial‐disease genes, causing dilated cardiomyopathy with ataxia (DCMA) and recessive Leber's hereditary optic neuropathy (LHON) or Leigh‐like disease, respectively (Davey et al., 2006; Stenton et al., 2022). Mutations in *DNAJC19* were first identified in patients with DCMA and 3‐methylglutaconic aciduria. The original genetic report mapped disease‐associated variants to the Pam18 homolog *DNAJC19* in multiple families (Davey et al., 2006). The second Pam18 homolog, DNAJC15 is expressed in selective tissues and hence the phenotype of DNAJC15 depletion is not as severe as for DNAJC19 (Strathdee et al., 2004). Epigenetic silencing in epithelial tissues influences cellular responses such as apoptosis upon chemotherapeutic treatment of cancer cells (Shridhar et al., 2001). Recently, biallelic pathogenic variants in DNAJC30 were discovered as cause of recessive LHON and have also been reported in patients with Leigh syndrome‐like presentations (Stenton et al., 2022). DNAJC30 is a nuclear‐encoded mitochondrial J‐domain protein implicated in complex I maintenance and repair and mutations in DNAJC30 lead to complex I deficiency and associates with the F_1_F_O_‐ATP synthase (Hatle et al., 2013; Tebbenkamp et al., 2018). Supporting a function of DNAJC30 for mitochondrial function, DNAJC30 knock out mice display reduced respiratory activity (Tebbenkamp et al., 2018). The matrix localized DNAJA3 has been linked to cancer and neurodegenerative disease. DNAJA3 isoforms contribute to cell survival pathways. The proapoptotic long isoform of DNAJA3 promotes cell death by facilitating cytochrome *c* release and activating mitochondrial caspases, whereas the antiapoptotic short isoform inhibits cytochrome *c* release and thereby suppresses apoptosis (Choi et al., 2012; Ng et al., 2014). DNAJA3 has been associated with multiple malignancies, including skin, breast, and colorectal cancers. It functions as an important tumor suppressor through its interactions with oncogenic factors such as ErbB2 and p53. In several cancers, DNAJA3 limits cell proliferation, motility, and invasion by suppressing EGFR and, further downstream, AKT signaling (Chen et al., 2009). Beyond oncology, DNAJA3 is also implicated in neurodegenerative disorders, including Parkinson's disease (Elwi et al., 2012). Additionally, a splicing mutation in murine DNAJC11 has been linked to motor neuron pathology with altered mitochondrial inner membrane architecture (Ioakeimidis et al., 2014; Violitzi et al., 2019).

## 8 CONCLUSION

J‐domain proteins fulfill various crucial functions for the biogenesis of mitochondrial proteins (Figure 3). Remarkably, they accompany mitochondrial proteins in every step of their biogenesis. J‐domain proteins bind to mitochondrial proteins when they are produced at ribosomes, are involved in their transport toward mitochondria, power the import of presequence‐containing proteins into the matrix, and modulate the assembly of respiratory chain subunits. J‐domain proteins also interact with cytosolic and nuclear protein aggregates that contain non‐imported mitochondrial precursor proteins and mediate transcriptional stress response. Most of these functions depend on their active J‐domain, revealing a cooperation with Hsp70 chaperones. However, the J‐domain proteins are more specific for substrates and functions and can bind to substrates independently of Hsp70. Understanding the role of this protein family for mitochondrial protein biogenesis and proteostasis will uncover fundamental processes that monitor biogenesis of mitochondrial proteins. Central questions still remain unclear. What is the role of J‐domain proteins for substrate‐specific targeting toward the mitochondrial surface? Do J‐domain proteins play a role in recently identified quality control mechanisms? How are J‐domain proteins dynamically segregated between partner proteins to functions in import and assembly of respiratory chain subunits? Addressing these questions will enable us to define central mechanisms of mitochondrial proteostasis.

## AUTHOR CONTRIBUTIONS

**Vitasta Tiku:** Visualization; writing – review and editing. **Georg Bossenz:** Visualization; writing – review and editing. **Janine Kirstein:** Conceptualization; writing – original draft; writing – review and editing; visualization. **Thomas Becker:** Conceptualization; writing – original draft; writing – review and editing; visualization.

## Data Availability

Data sharing not applicable to this article as no datasets were generated or analysed during the current study.
